# Physicochemical Properties and *in vitro* Digestibility of Myofibrillar Proteins From the Scallop Mantle (*Patinopecten yessoensis*) Based on Ultrahigh Pressure Treatment

**DOI:** 10.3389/fnut.2022.873578

**Published:** 2022-04-11

**Authors:** Xiaohan Liu, Kemin Mao, Yaxin Sang, Guifang Tian, Qiuyue Ding, Wenyi Deng

**Affiliations:** College of Food Science and Technology, Hebei Agricultural University, Baoding, China

**Keywords:** ultrahigh pressure, myofibrillar protein, scallop mantle, *in vitro* digestibility, structural properties

## Abstract

The utilization of myofibrillar proteins (MPs) from the scallop mantle was limited due to its poor digestibility *in vitro*. In this study, structural properties and *in vitro* digestibility of MP were evaluated after modified by ultra-high pressure (UHP) at different pressures (0.1, 100, 200, 300, 400, and 500 MPa). The results showed that high pressure could significantly increase the ordered structure content like α-helix, inhibit the formation of disulfide bonds, and decrease surface hydrophobicity. Moreover, MP possessed the optimal solubility and *in vitro* digestibility properties at 200 MPa due to the minimum particle size and turbidity, relatively dense and uniform microstructure. The results indicated that the UHP treatment was an effective method to improve the digestibility of MP from scallop mantle and lay a theoretical basis for the functional foods development of poor digestion people and comprehensive utilization of scallop mantles.

## Introduction

In recent years, the annual output of the highly economical scallops (*Patinopecten yessoensis*) has reached nearly 2 million tons in China. As the main byproduct of scallop processing, the annual production of scallop mantle is also on the increase, which was good resource for lowering blood lipids, anti-aging, and resisting atherosclerosis due to its abundant nutrients such as proteins, polysaccharides, taurine, essential amino acids, and mineral elements (1). Myofibrillar protein (MP) is salt-soluble and accounts for 55–65% of the total protein content of scallop mantle, which directly affects the characteristics of solubility, *in vitro* digestibility properties, and emulsification properties of scallop byproducts (2). However, most scallop mantles were randomly discarded and wasted (3), and only a small portion was reprocessed partly due to poor solubility and low utilization rate of MP. Therefore, it is particularly necessary to improve the solubility and *in vitro* digestibility of MP to explore function foods for poor digestion people and increase the utilization rate of the scallop mantle (4).

The ultra-high pressure (UHP) treatment is an alternative to usual thermal processes (5), which is related to flavor, structural, and functional modification of food-derived components like protein. During UHP (100–1,000 MPa), the hydration of the protein chains might be formed through the liquid pressure transmission medium followed by the behavior changes of protein like denaturation, unfolding, and aggregation. Therefore, the UHP treatment was applied to reduce particle size, increase solubility, improve texture, and functional properties of food-derived proteins (6). UHP treatment might cause variable alterations in MP structure by protein unfolding and aggregation. It is bound up with the exposure of protein side chain amino acids like Phe, Tyr, Trp, Lys, and Arg, which were the target cleavage sites of pepsin or trypsin determining the extents of protein digestion. The solubility of MP from pork meat increased when it was treated under appropriate UHP treatment (≤400 MPa) but decreased under over high pressure (>400 MPa). It could be attributed to the fact that the water amount of MP might increase after proper UHP treatment while the water of MP might be redistributed to the outer compartment of MP under high pressure (7). UHP treatment could modify the secondary structure of oyster protein and enhance its solubility and digestibility. Compared with the untreated one, the digestibility of the treated samples (500 MPa) increased from 26.3 to 39.5% in the stomach and from 62.1 to 83.7% in the total digestion process (8). Zhang et al. stated that MP from chicken breast meat had maximum solubility and gel hardness, minimum particle size, as well as dense and uniform microstructure after the UHP treatment at 200 MPa due to the massive solubilization of myosin heavy chain and actin, and the exposure of both tyrosine and tryptophan residues (6). Previous study showed that high-pressure processing caused MP from bovine *longissimus dorsi* muscle meat disorganization, affected protein digestion kinetics *in vitro*, and changed muscle structure, which correspondingly improved its solubility and *in vitro* digestibility at 600 MPa (9). Although relevant research has focused on the modification of MP structure and function under UHP treatment, knowledge of the effect of UHP treatment on MP from scallop mantle was limited especially for the relationship between the structure and *in vitro* digestibility. Therefore, it is necessary to explore the change mechanism of physicochemical properties and *in vitro* digestibility of MP from the scallop mantle (*Patinopecten yessoensis*) during the UHP treatment.

The aim of the study was to explore the effect of UHP treatment on the solubility and *in vitro* digestibility of MP from scallop mantle by clarifying the relationship between the structure and function. After treated at different pressures (100, 200, 300, 400, and 500 MPa), respectively, the size, zeta potential, surface hydrophobicity, sulfhydryl groups, and microstructure of MP from scallop mantles were detected. Moreover, the influences of the structure change on solubility and *in vitro* digestion were elucidated. The results of this study would broaden the application of MP from the scallop mantle, improve the utilization rate of the scallop mantle, and lay the theoretical foundation for the further development of easily digestible products.

## Materials and Methods

### Materials

Scallop (*Patinopecten yessoensis*) was purchased from an aquatic product market (Qinhuangdao, Hebei, China). All samples were immediately transported on ice to the laboratory. And then they were stored at 4°C and used within 48 h. Protein marker (PR 10–250 kDa) and 5 × Sodium dodecyl sulfate-polyacrylamide gel electrophoresis (SDS-PAGE) loading buffer were obtained from Sevenbio Co. Ltd. (Sevenbio, Beijing, China). Disodium hydrogen phosphate, sodium dihydrogen phosphate, ethylenediaminetetraacetic acid (EDTA), sodium chloride, urea, 5,5'-dithiobis-(2-nitrobenzoic acid) (DTNB), sodium dodecyl sulfate (SDS), 1-anilino naphthalene-8-sulfonate (ANS), Coomassie Brilliant Blue G-250, and bovine serum albumin (BSA) were purchased from the Sigma Reagent Co. Ltd. (St. Louis, MO, USA). All chemical reagents used in this study were analytical or chromatographic grade.

### Extraction of MPs From Scallop Mantle

Myofibrillar protein was extracted from the scallop mantle as described by Zhang et al. with few modifications (10). Buffer A was 20 mmol/L phosphate buffer at PH 7.0 containing 100 mmol/L NaCl and 1 mmol/L EDTA while Buffer B was 25 mmol/L phosphate buffer at PH 7.0 containing 0.6 mmol/L NaCl. The scallop mantle was cut into small pieces, added to buffer A at a ratio of 1:10 (W/V), homogenized in ice bath at 10,000 rpm for 90 s, centrifuged (4°C) at 6,790 *g* for 20 min to collect the precipitates, and extracted the precipitate again with buffer A. The entire process was repeated twice. The precipitates were added to a certain amount of buffer B, homogenized at 10,000 rpm for 20 s and centrifuged (4°C) at 6,790 *g* for 20 min. Finally, the supernatant (i.e., MP) was collected after centrifugation. The MP contents were determined by the Biuret method using BSA as standard (11).

### Ultra-High Pressure Treatment of MP

The MP solutions sealed in polyethylene bags at 6°C were placed in the chamber of ultra-high pressure processor (HPP.L2-600-2, Hua tai Ltd., Tianjin, China). The high pressure increased to the designed pressure (100, 200, 300, 400, and 500 MPa) at a rate of 3.5 MPa/s and maintained for 10 min, respectively.

### Sodium Dodecyl Sulfate-Polyacrylamide Gel Electrophoresis

The SDS-PAGE experiment was carried out according to the method of Laemmli (12) with a few modifications. Diluted MP solutions (1 mg/ml) were mixed with 5 μl SDS-PAGE loading buffer (5×) and heated for 5 min at 100°C. Then, 20 μl MP solutions were electrophoresed on a 10% separating gel and a 5% stacking gel. Finally, a constant current of 80 V was employed for the separation gel until all samples were input into the stacking gel while 120 V was used for the stacking gel until the indicator was about 5 mm above the gel edge. After electrophoresis, the gels were stained by Coomassie brilliant Blue for 1 h and subsequently de-stained with 5% methanol and 7.5% acetic acid. The gels were scanned and analyzed on a gel imager (Tanon-4600SF, Tanon Ltd., Shanghai, China) after decolorizing. MP was identified with standard (Protein Ladder 10–250 kDa).

### Circular Dichroism

The secondary structure proportion of MP solutions was recorded according to the method of Wu et al. using CD spectrometer (Applied Photophysics Ltd, JASCO810, UK) with a quartz cell of 1 cm optical path in the wavelength range of 195–260 nm (13). The step size of MP (0.2 mg/ml) measurement was 1 nm, and the scanning speed was 50 nm/min. Protein secondary structures were determined as percentages of α-helix, β-sheet, β-turn, and random coil using the Alix's method.

### Endogenous Fluorescence Spectra

The endogenous fluorescence spectrum was obtained by the method described by Jia et al., with few modifications (14). MP (0.5 mg/ml) was detected using the fluorescence spectrophotometer (F-320, Gangdong Instruments Ltd., Tianjin, China) at an excitation wavelength of 295 nm and emission wavelength of 300–420 nm. The constant crack width between excitation and emission wavelength was 10.0 nm.

### Surface Hydrophobicity

The surface hydrophobicity was obtained by the method described by Jiang et al., and with slight modifications (15). After mixing 5 ml MP containing 0, 0.2, 0.4, 0.6, 0.8, and 1.0 mg/ml protein with 25 μl ANS solution (8 mmol/L ANS in 20 mmol/L phosphate buffer, pH 7.5), the mixtures were placed in the dark at room temperature for 25 min. The relative fluorescence intensity at the excitation wavelength of 374 nm and the emission wavelength of 485 nm was recorded with the fluorescence spectrophotometer. The slope between the fluorescence intensity and the protein concentration was represented the surface hydrophobicity of MP.

### Total and Reactive Sulfhydryl Contents

The total sulfhydryl contents of the MP were determined according to the previous report with slight modifications (16). A total of 0.5 ml MP (1 mg/ml) was mixed with 4.5 ml of solution containing 8 mol/L urea and 10 mmol/L EDTA (pH 6.0), and 100 μl Ellman's reagent (10 mmol/L DTNB in 0.1 mol/L NaH_2_PO_4_ buffer). Then it was set in the dark at room temperature for 25 min and measured at 412 nm with ultraviolet-visible spectrophotometer (N5000, Yoke Analysis Instrument Co., Shanghai, China). The supernatants without DTNB were used as the control.

The reactive sulfhydryl contents were obtained by the method described of Guo et al., and with slight modifications (17). A total of 5 ml of MP (1 mg/ml) was mixed with 20 μl of DTNB. Then, the solutions were kept at room temperature for 1 h and measured at 412 nm with ultraviolet-visible spectrophotometer. Sulfhydryl concentration was calculated using the following equation:


(1) $$\begin{array}{l} Sulfhydryl\;concentration\;\left(\mu \;mol/g\;protein\right)\;\\ =\frac{A412-A412r}{k\times c}\times 1,000 \end{array}$$

where A_412r_ and A_412_ are the absorbance of reagent blank and sample at 412 nm, k is the extinction coefficient (13,600 M^−1^ cm^−1^), and c is the protein concentration of samples.

### Particle Size and Zeta Potential

Malvern Zeta sizer Nano ZS90 instrument (Malvern Instruments Ltd., Malvern, England) was applied to determine the particle size distribution and zeta potential of the MP solutions (0.5 mg/ml).

### Atomic Force Microscopy Measurements

The morphology of the MP was monitored by the atomic force microscopy instrument (MFP-3D infinity, Oxford Instruments Ltd., UK) through a previously described method with slight modifications (18). MP was diluted to 10 ppm, then placed on glass slide and air-dried at room temperature.

### Solubility and Turbidity Measurements

Solubility was measured using the method with slight modifications (19). MP (3 mg/ml) was centrifuged at 6,790 *g* for 15 min. The supernatant was collected and measured by the biuret method, using BSA as a standard. The formula of the protein solubility was using the following equation:


(2) $$\begin{array}{l} Protein\;solubility\;\left(\%\right)\;\\ =\frac{protein\;content\;of\;supernatant\left(mg\right)}{total\;protein\;content\;in\;solution\;\left(mg\right)}\;\times \;100\% \end{array}$$

Turbidity was measured using the modified protocol with slight modifications (20). MP (1 mg/ml) was incubated for 30 min at room temperature and measured at 340 nm with an ultraviolet-visible spectrophotometer with buffer B as the blank.

### *In vitro* Digestion

*In vitro* digestion was measured using the method with slight modifications (21). Due to the short residence in the oral cavity, the samples were mainly subjected to gastric and small intestinal stages to simulating digestion *in vitro*.

### Statistical Analysis

In this study, statistical analyses of the data were performed by using SPSS 22.0 (SPSS 22.0, Chicago, USA) and *post-hoc* Duncan's test of ANOVA test (significant differences). All experiments were measured in triplicate and data were expressed as the average ± standard deviation (*SD*). Results were considered significantly different for *p* ≤ 0.05 (22).

## Results and Discussion

### Effect of UHP Treatment on Molecular Structures of MP From Scallop Mantle

#### Primary Structure

**Figure 1:**
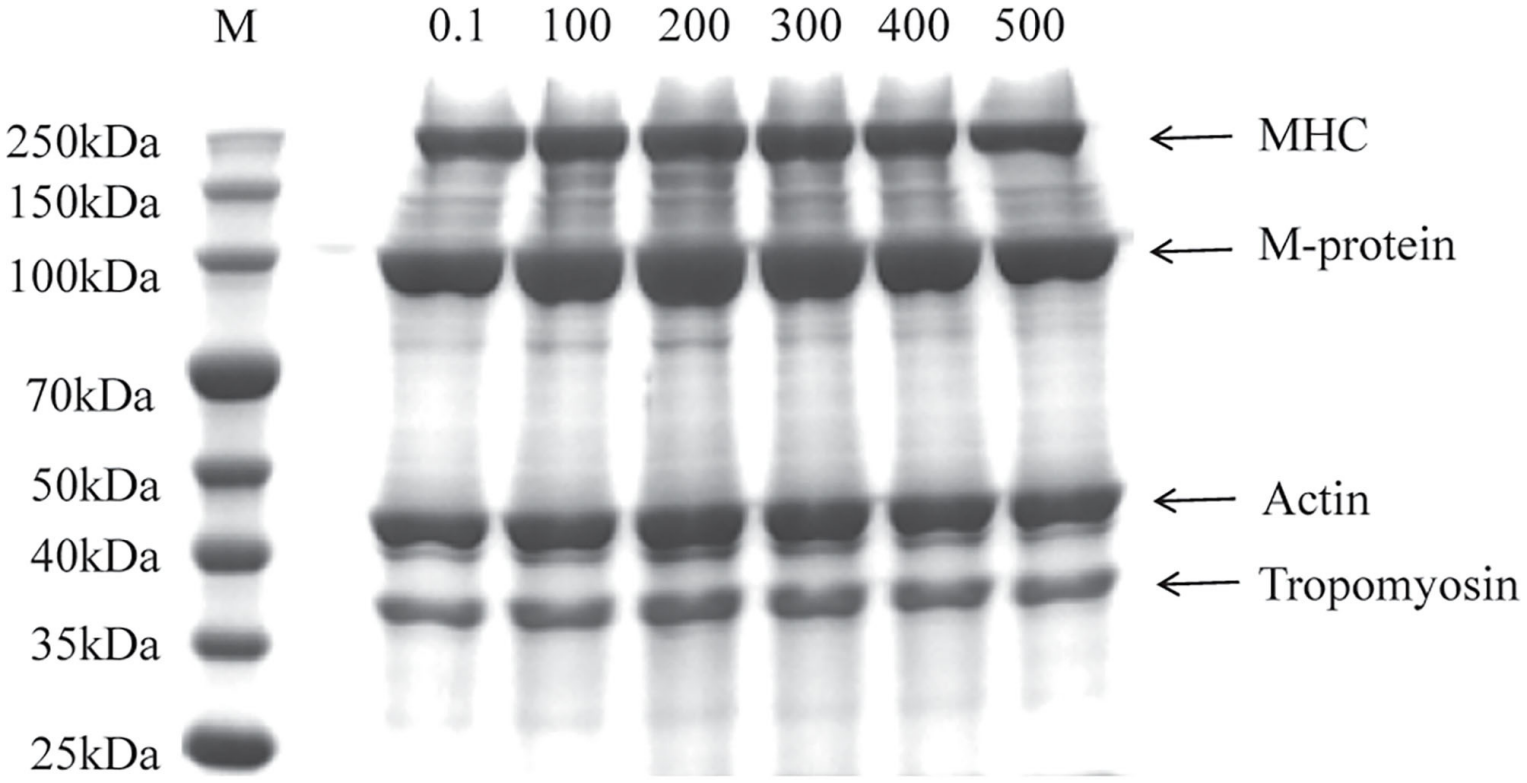
Sodium dodecyl sulfate-polyacrylamide gel electrophoresis (SDS-PAGE) profile of myofibrillar protein (MP) from the scallop mantle at different pressures (Lane M: protein marker, MHC: myosin heavy chain).

The SDS-PAGE was performed to visualize the change of primary structure in different MP from the scallop mantle. As shown in Figure 1, all samples showed the typical molecular pattern distribution for MP with, myosin heavy chain (MHC, 220 kDa), M-protein (97 kDa), actin (44 kDa), and tropomyosin (37 kDa), which was similar to Peng et al. (7). There was no significant difference in the bands between different samples, which demonstrated that the UHP treatment could not change the molecular weight of the protein and degrade the MP molecules.

#### Secondary Structure

Circular dichroism was used to assess the change of secondary structure in different samples. As shown in Table 1, the relative content of α-helix, β-turn, and random coil significantly increased after the UHP treatment, while the β-sheet content significantly decreased. The β-sheet relies on the hydrogen bonds between peptide bonds. That's probably because the hydrogen bonding was weakened with the increase of the pressure resulting from the unfolding of protein (23). Inter hydrogen bonds of the peptide chain were exhibited the stability capacity of α-helium and were relied on by β-plates. It can be attributed to the fact that the cavitation force produced by the UHP treatment, could disrupt the interaction between protein molecules. It could be concluded that UHP treatment was an effective method to significantly increase the ordered structure contents and stable conformation of MP.

**Table 1:** Secondary structure contents of myofibrillar protein (MP) from the scallop mantle treated by ultra-high pressure (UHP).

**UHP–treatment (MPa)**	**Secondary structure (%)**			
	**α-helix**	**β-sheet**	**β-turn**	**Random coil**
0.1	9.80 ± 0.15 ^a^	40.60 ± 0.19 ^a^	19.28 ± 0.14 ^a^	33.20 ± 0.17 ^a^
100	10.49 ± 0.13 ^b^	38.73 ± 0.20 ^b^	19.52 ± 0.21 ^ab^	33.28 ± 0.18 ^ab^
200	11.02 ± 0.29 ^c^	38.40 ± 0.15 ^c^	19.27 ± 0.13 ^a^	33.43 ± 0.20 ^ab^
300	12.20 ± 0.15 ^d^	36.29 ± 0.15 ^d^	19.67 ± 0.20 ^b^	33.57 ± 0.19 ^b^
400	11.77 ± 0.19 ^d^	35.70 ± 0.17 ^e^	19.50 ± 0.15 ^ab^	33.91 ± 0.08 ^c^
500	11.71 ± 0.17 ^d^	35.30 ± 0.15 ^f^	20.08 ± 0.15 ^c^	34.21 ± 0.20 ^c^

*Different letters (a–f) in the same column means significant differences (p < 0.05) among samples treated under different pressures*.

#### Tertiary Structure

**Figure 2:**
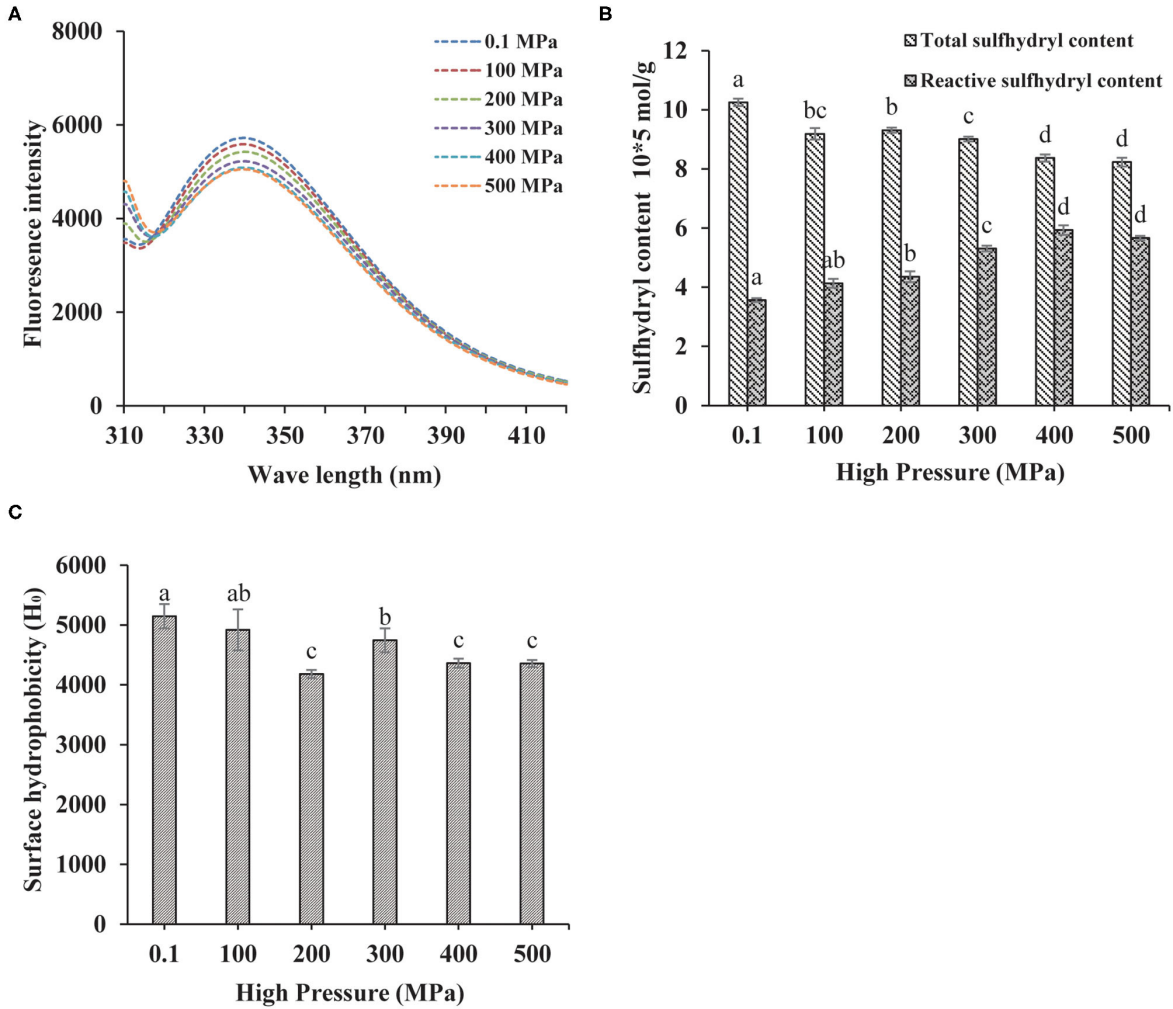
Changes in the endogenous fluorescence **(A)**, sulfhydryl contents **(B)**, and surface hydrophobicity **(C)** of MP from the scallop mantle under the ultra-high pressure (UHP) treatment.

The fluorescence spectrum was applied to reflect the tertiary structure information of MP. The 295 nm was selected as the excitation wavelength to detect the fluorescence intensity caused by the conformational changes from the tryptophan, phenylalanine, and tyrosine residues (24). Higher the pressure, lower the intrinsic fluorescence emission spectra as shown in Figure 2A, indicating that the tertiary structure of the protein was changed by UHP-treatment. Tryptophan, phenylalanine, and tyrosine residues were exposed to the external polar environment under strong mechanical forces and the cavitation produced by the UHP-treatment, therefore, reducing the endogenous fluorescence (15).

#### Total and Reactive Sulfhydryl Contents

Protein sulfhydryl existed in the form of free sulfhydryl and disulfide bonds. The protein structure and interactions were affected by conformation changes between free sulfhydryl and disulfide bonds. Proteins would aggregate if disulfide bonds were formed during the UHP treatment, which was related to the increase of surface hydrophobicity (25). As shown in Figure 2B, the total sulfide contents of MP solution decreased with the increase of pressure. The disulfide bonds were liable to form owing to the exposure of sulfhydryl from the protein interior and the shortened distances between intermolecular sulfhydryl contents (2). Proper UHP treatment (150–300 MPa) would facilitate to the formation of disulfide bonds and the decrease of sulfhydryl contents (26). As seen in Figure 2B, the contents of reactive sulfhydryl increased with the increase of pressure. It might be ascribed to the exposure of the internal sulfhydryl, resulting from the separation of subunits and breakage of the disulfide bonds. In addition, it might be due to the stretching and unfolding of protein molecules that exposed the interior sulfhydryl under UHP treatment (27).

#### Surface Hydrophobicity

Besides directly related to the solubility and *in vitro* digestion of proteins, the surface hydrophobicity was a key index to show the extent of surface hydrophobic residues on the surface of protein molecules (14). Compared with the control (0.1 MPa), the surface hydrophobicity of the treated samples decreased significantly as shown in Figure 2C. Hydrophobic amino acid residues generally appear obscured deep in the folded structure of proteins. After the UHP treatment, the protein conformation structure became loose and destabilized due to the stretching and unfolding of MP, which was detrimental to the hydrophobic interactions between hydrophobic ANS probes and proteins (28). Surface hydrophobicity got its minimum at 200 MPa. When the pressure was higher than 300 MPa, the hydrophobic groups were exposed to the outer of the protein from the interior, which was beneficial for the binding of ANS.

### Effect of UHP Treatment on Dispersion Behavior of MP From Scallop Mantle

#### Particle Size and Zeta Potential Distribution

**Figure 3:**
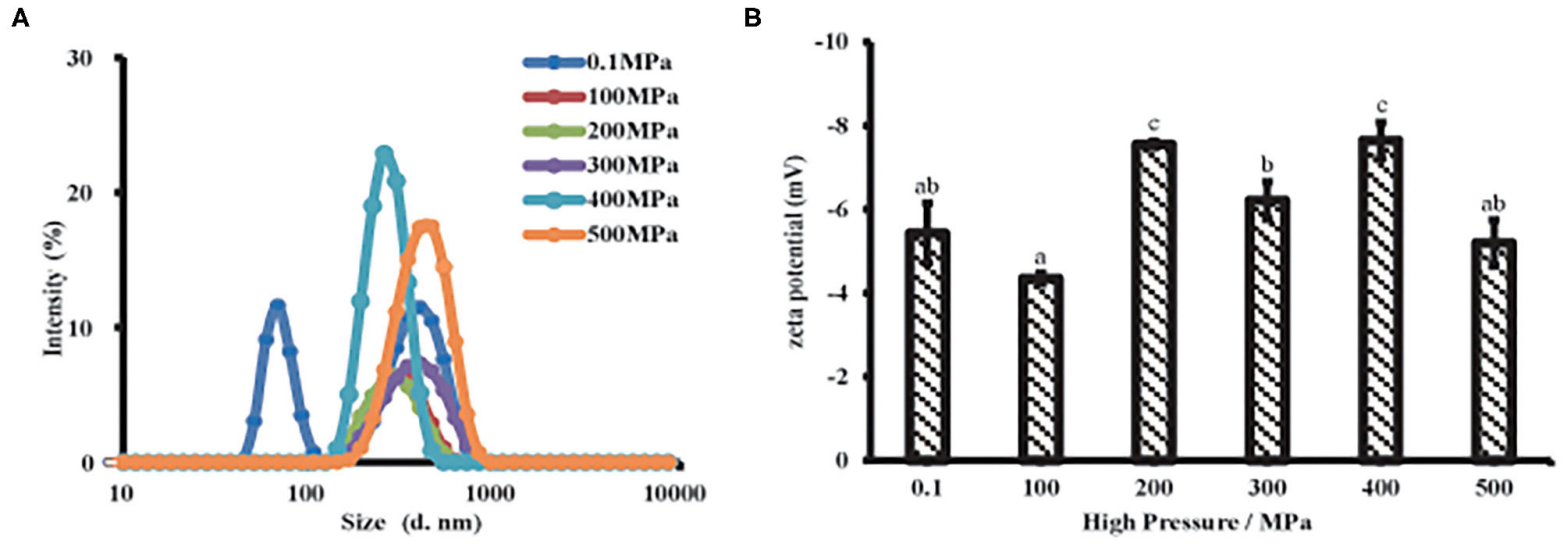
Particle properties of MP solution from the scallop mantle under the UHP treatment. Particle size **(A)** and zeta potential **(B)**.

Particle size was a key factor that characterized the proteins aggregation and affected proteins solubility. As shown in Figure 3A and Supplementary Table S1, the untreated samples exhibited a bimodal and broader particle distribution, while MP samples had a unimodal and narrow distribution after UHP treatment. The particle size of MP presented a significantly decrease under the UHP treatment and achieved the minimum at 200 MPa. Initial protein polymer was broken into small particles by the violent agitation during UHP process. It had a close relation to the denaturation of protein molecules and the rupture of non-covalent bonds caused by the separation of muscle proteins, depolymerization of actin and myosin. However, the particle size increased when the pressure was over 200 MPa, indicating that protein polymers were formed again resulting from intermolecular desulfurization bridges and hydrophobic interactions as previous studies reported (6). The results showed that MP would undergo interruption and depolymerization during UHP treatment.

Zeta potential was related to the dotted residues located or near the surface of the suspended particle, which was closely linked to proteins dispersibility and aggregation (29). All samples were exhibited a negative charge (Figure 3B) due to the negatively charged amino acid residues like glutamic acid at neutral PH (18). After the UHP treatment, the absolute zeta potential increased, meaning more dotted side chains were exposed to the surface of the dispersed particles, which was beneficial to improve the solubility and *in vitro* digestibility of MP. With respect to the untreated sample, a high absolute zeta potential was presented in treated ones except 100 MPa, which means particle aggregation was easily formed and hard to separate because the electrostatic repulsion between the protein particles was weak (30). The samples possessed a high absolute zeta potential after UHP treatment at 200 and 400 MPa, respectively. The results were consistent with the particle size. Small protein particles had more charge sites exposed on the suspended particles than big ones because of the bigger surface area. As is known that the high absolute zeta potential on protein particles could strengthen the electrostatic repulsion between particles, which might give rise to an increase in solubility and a decrease in protein aggregation (31). Therefore, the higher absolute zeta potential, the protein particles had (~8 mV) after the UHP treatment, the stronger electrostatic repulsion existed among the protein particles, and the higher the MP solubility. The UHP treatment of 200 MPa endowed MP, the minimum particle size and the maximum absolute zeta potential as described in Figures 3A,B, which might promote the dissolution of MP solutions (29).

#### Microstructure

**Figure 4:**
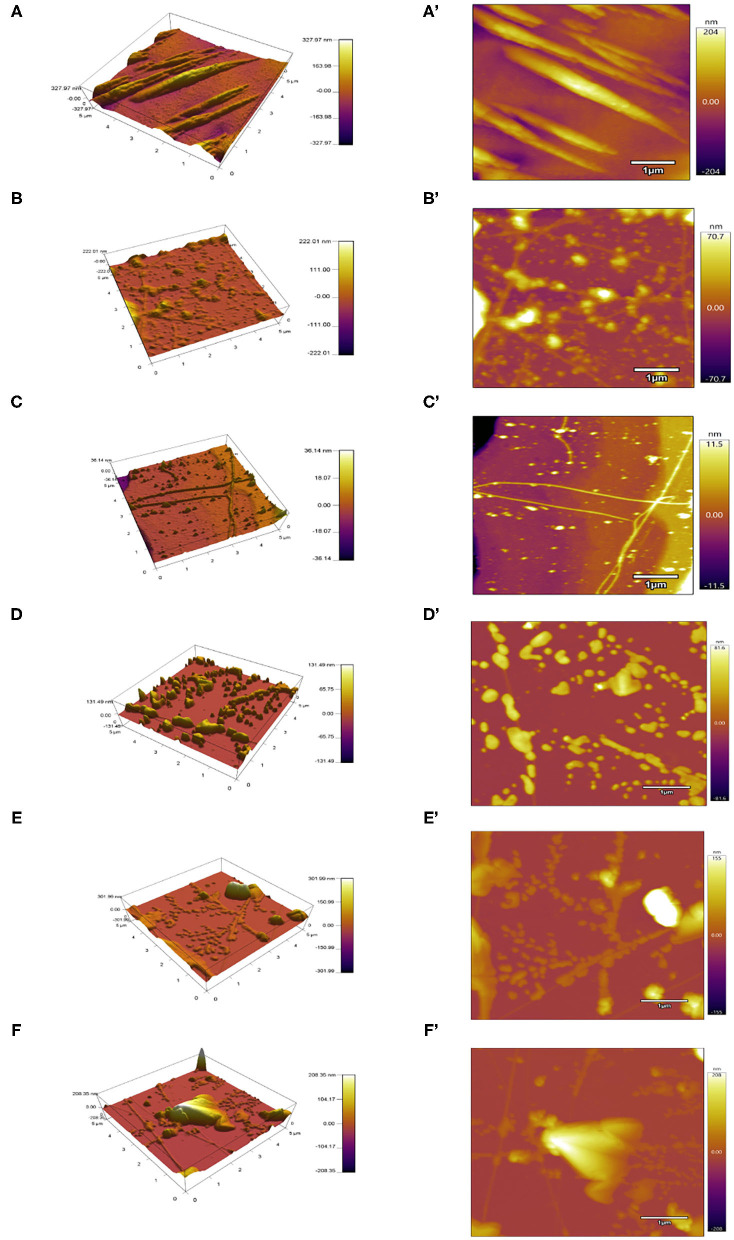
Atomic force microscopy (AFM) images of different UHP treated MP solutions from the scallop mantle. 3D view **(A–F)** and top view **(A'–F')**.

The microstructure plays an important role in the functional properties of proteins. AFM was a common technical means to characterize the MP microstructures (32). As displayed in Figure 4, the untreated protein particles gathered with a mainly rough and non-homogeneous morphology, and the height of around 327 nm. MP treated below or equal to 200 MPa had a dense and homogeneous network with many filaments and irregular cavities, especially at 200 MPa. With the UPH treatment over 200 MPa, MP exhibited a large and heterogeneous structure due to the MP denaturation excessively and exposure of interior hydrophobic and sulfhydryl groups. It also was related to the relative speed of protein unfolding and aggregation. When the speed of protein aggregation was faster than that of unfolding, the dense and uniform structure would be formed, and vice versa (6).

#### Solubility and Turbidity

**Figure 5:**
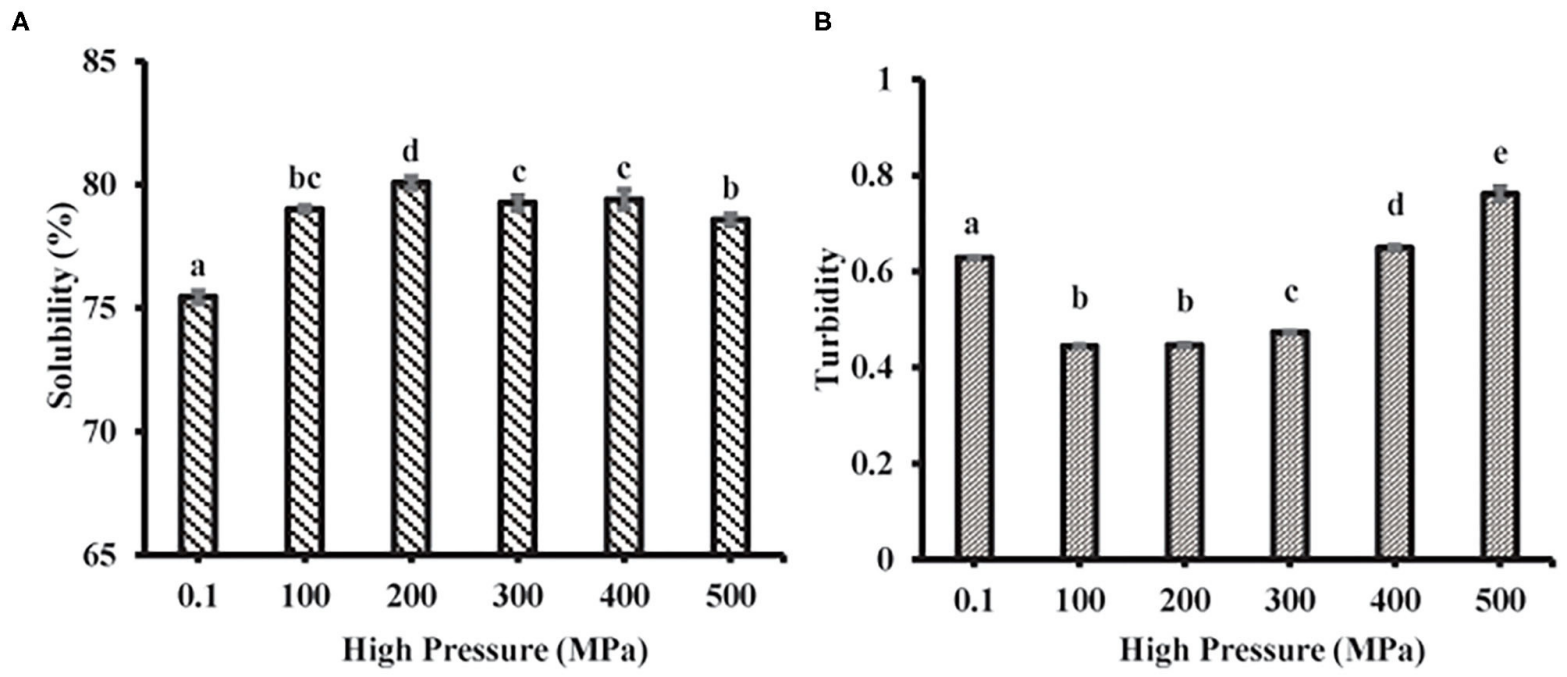
Solubility **(A)** and turbidity **(B)** of MP from the scallop mantle under the UHP treatment.

Solubility mainly reflects the denaturation and polymerization of protein while turbidity reflects the degree of protein aggregation. The MP solubility of the scallop mantle significantly increased after UHP treatment and reached the highest level at 200 MPa (Figure 5A). It might be induced by the depolymerization of actin and actomyosin under UHP treatment. It was in accordance with the previous report that the increase of protein solubility resulted from the increase of shear treatment, which could disrupt newly formed polymers and prompt the interaction of unfolded protein and water molecules (33). The solubility reduction of MP from scallop mantle might be attributed to the fact that high-pressure treatment (≥300 MPa) promoted the formation of insoluble protein aggregates through non-disulfide and disulfide bonds relying on the exposure of the interior hydrophobic resides and sulfhydryl groups (34). The results were consistent with a previous study that the solubility of MP from beef muscle increased at 200 MPa but deceased when the pressure was over 200 MPa (34). Moreover, it could be speculated from the data of particle size and solubility that the MP solubility was inversely proportional to its particle size because small particle size was beneficial to the protein–water interaction (35).

The turbidity changes of MP samples after different UHP treatments were shown in Figure 5B. UHP-treatment decreased the turbidity of MP solution when the pressure was below 400 MPa. The turbidity results were also in agreement with the tendency of particle size (Figure 3A) due to high shear energy waves. The turbidity decrease (≤300 MPa) demonstrated that the decrease of protein aggregation. When the pressure was over 300 MPa, the turbidity increased with the increase of pressure caused by the myosin aggregation after the unfolding of the α-helix. The results were in accordance with the previous results on the particle size of MP solution, which indicated that the decrease of protein aggregation was the main reason for the increase of protein solubility (35).

### Effect of UHP Treatment on *in vitro* Digestion of MP From Scallop Mantle

**Figure 6:**
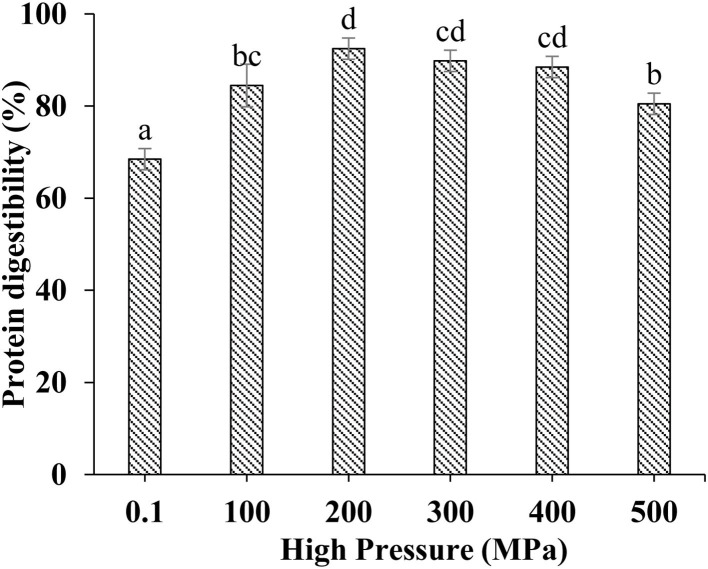
*In vitro* digestibility of MP from the scallop mantle treated by the UHP treatment.

Protein digestibility was an important indicator to evaluate the nutritional value of food protein, especially for poor digestion people. The *in vitro* digestibility changes of different treated MP samples were shown in Figure 6. UHP could enhance the MP of scallop mantle *in vitro* digestibility. And 200 MPa was the best pressure. The *in vitro* digestibility of MP increased from 68.47 ± 2.31% to 92.47 ± 2.30% at 200 MPa. The digestibility was associated with the spatial structure of proteins under different UHP treatment (36). The extents of protein digestion in the gastrointestinal tract depends on the contents of Phe, Tyr, Trp, Lys, and Arg, the target cleavage sites of pepsin or trypsin, which was affected by the exposure of amino acids in protein side chain through protein unfolding and aggregation during UHP treatment. Ultrahigh pressure would break the chemical bonds and forces like hydrogen bonds, disulfide bonds, hydrophobicity, and Van der Waals forces as forementioned (37). The results were consistent with particle size, surface hydrophobicity, and solubility (38).

## Conclusions

The present study demonstrated that the UHP treatment had apparent impacts on the physicochemical properties and digestibility of MP. The UHP treatment was an effective method to exposing interior hydrophobic and sulfhydryl contents. The digestibility *in vitro* of MP was negatively correlated to its surface hydrophobicity, particle size, and turbidity but positively correlated to its solubility. The 200 MPa was the optimum pressure to improve solubility and *in vitro* digestibility of MP from scallop mantle. Since the effect of UHP treatment on physicochemical properties and digestibility of MP were revealed, studies are underway to investigate the digestible scallop products. It would lay a scientific theoretical foundation for the development of digestible scallop products and comprehensive utilization of scallop mantle MPs.

## Data Availability Statement

The original contributions presented in the study are included in the article/Supplementary Material, further inquiries can be directed to the corresponding author.

## Author Contributions

XL: conceptualization, data curation, formal analysis, methodology, and writing–original draft. KM: methodology, conceptualization, and software. YS: supervision, funding acquisition, and writing–review and editing. GT: conceptualization, supervision, and writing–review and editing. QD: methodology and data curation. WD: methodology and writing–original draft. All authors contributed to the article and approved the submitted version.

## Funding

This study was supported financially by the National Key R&D Program of China (2019YFD0902003).

## Conflict of Interest

The authors declare that the research was conducted in the absence of any commercial or financial relationships that could be construed as a potential conflict of interest.

## Publisher's Note

All claims expressed in this article are solely those of the authors and do not necessarily represent those of their affiliated organizations, or those of the publisher, the editors and the reviewers. Any product that may be evaluated in this article, or claim that may be made by its manufacturer, is not guaranteed or endorsed by the publisher.
