# Supplementary material for: Physicochemical Properties and in vitro Digestibility of Myofibrillar Proteins From the Scallop Mantle (Patinopecten yessoensis) Based on Ultrahigh Pressure Treatment
Source: Front Nutr. 2022 Apr 11;9:873578. doi: 10.3389/fnut.2022.873578 (PMC9037751; doi:10.3389/fnut.2022.873578)
Supplement: Supplementary file 1 [file Table_1.docx]

**Table S1** Effects of non-pressurized and pressurized on the average particle size of MP.

| UHP - treatment (MPa) | Average particle size ± SD (nm) |
| --- | --- |
| 0.1 | 324.30 ± 12.99 ^a^ |
| 100 | 331.03 ± 7.38 ^a^ |
| 200 | 295.30 ± 3.82 ^b^ |
| 300 | 309.26 ± 21.28 ^ab^ |
| 400 | 328.30 ± 22.91 ^a^ |
| 500 | 329.97 ± 5.18 ^a^ |

Notes：Different letters (a-b) in the same column means significant differences (p < 0.05) among samples treated under different pressures.
